# The Strong Anti-Kinetoplastid Properties of Bee Propolis: Composition and Identification of the Active Agents and Their Biochemical Targets

**DOI:** 10.3390/molecules25215155

**Published:** 2020-11-05

**Authors:** Godwin U. Ebiloma, Nahandoo Ichoron, Weam Siheri, David G. Watson, John O. Igoli, Harry P. De Koning

**Affiliations:** 1 School of Health and Life Sciences, Teesside University, Middlesbrough TS1 3BX, UK; g.ebiloma@tees.ac.uk; 2 Phytochemistry Research Group, Department of Chemistry, University of Agriculture, Makurdi 2373, Nigeria; nannchoron@gmail.com (N.I.) igolij@gmail.com (J.O.I.); 3 Strathclyde Institute of Pharmacy and Biomedical Sciences, University of Strathclyde, Glasgow G1 1XQ, UK; weamsiheri@gmail.com (W.S.), d.g.watson@strath.ac.uk (D.G.W.); 4 Institute of Infection, Immunity and Inflammation, College of Medical, Veterinary and Life Sciences, University of Glasgow, Glasgow G12 8TA, UK

**Keywords:** propolis, mode-of-action, *Trypanosoma*, Leishmania, Crithidia, kinetoplastid, natural compound, drug discovery

## Abstract

The kinetoplastids are protozoa characterized by the presence of a distinctive organelle, called the kinetoplast, which contains a large amount of DNA (kinetoplast DNA (kDNA)) inside their single mitochondrion. Kinetoplastids of medical and veterinary importance include *Trypanosoma* spp. (the causative agents of human and animal African Trypanosomiasis and of Chagas disease) and *Leishmania* spp. (the causative agents of the various forms of leishmaniasis). These neglected diseases affect millions of people across the globe, but drug treatment is hampered by the challenges of toxicity and drug resistance, among others. Propolis (a natural product made by bees) and compounds isolated from it are now being investigated as novel treatments of kinetoplastid infections. The anti-kinetoplastid efficacy of propolis is probably a consequence of its reported activity against kinetoplastid parasites of bees. This article presents a review of the reported anti-kinetoplastid potential of propolis, highlighting its anti-kinetoplastid activity in vitro and in vivo regardless of geographical origin. The mode of action of propolis depends on the organism it is acting on and includes growth inhibition, immunomodulation, macrophage activation, perturbation of the cell membrane architecture, phospholipid disturbances, and mitochondrial targets. This gives ample scope for further investigations toward the rational development of sustainable anti-kinetoplastid drugs.

## 1. Introduction

Kinetoplastids are a diverse group of flagellated protozoa, whose common feature is the presence of a structure of mitochondrial DNA located at the base of the flagellum, called the kinetoplast. *Trypanosoma* and *Leishmania* species are the kinetoplastids known to cause disease in humans, as well as in livestock and/or companion animals such as dogs. The most common human diseases caused by these parasitic protozoa are Human African Trypanosomiasis (HAT or sleeping sickness), caused by *Trypanosoma brucei* subspecies *T. b. rhodesiense* and *T. b. gambiense*, American Trypanosomiasis (Chagas disease), caused by *T. cruzi*, and several forms of leishmaniasis (cutaneous, mucocutaneous, and visceral) caused by an estimated 20 different *Leishmania* species. They are designated neglected tropical diseases (NTDs) by the World Health Organization (WHO) [1]. Over one billion people from tropical and subtropical regions of the world are at risk of this group of vector-borne kinetoplastid diseases [2,3].

Vaccines have not yet been developed and are unlikely to be developed in the foreseeable future, while interest in drug discovery and development for these diseases is low, primarily because the populations (mostly poor and low-income) affected by these diseases do not represent a profitable market for the pharmaceutical industry [2]. This task is made even more daunting by differing clinical manifestations of the various forms of leishmaniasis and trypanosomiasis, thus requiring different pharmaceutical and pharmacokinetic requirements for each drug to be used against each form of the infection [4,5].

Chemotherapeutic options currently in use do not give optimal results due to high toxicity, damaging side effects, long periods of treatment, and drug resistance [2,5,6,7,8]. However, WHO’s collaboration with the pharmaceutical industry and other stakeholders to accelerate research and development of new treatments for NTDs [9] has kindled interest in research toward developing drugs with minimal side effects and higher efficacy [4].

The extensive and continuous use of natural products in folk medicine is evidence that they contain bioactive molecules that can be developed into drugs. They provide a rich source of molecules with structural and chemical diversity that can serve as drugs or scaffolds for the development of new drugs [10,11]; as a result, there is a rapidly growing interest in natural product-based drug approaches [12].

Owing to its long use in traditional medicine for treatment of infectious diseases and its reported antimicrobial activities [13,14], the chemical compositions and properties of propolis from diverse locations and floral origins are now being intensely investigated. Many scientific studies on propolis samples from different botanical sources, geographical regions, and seasons of collection have reported its pharmacological activities with results that point to its therapeutic potential against diseases caused by kinetoplastids [14,15,16,17,18,19,20,21,22,23,24]. This review contributes to this growing body of knowledge by bringing together the chemistry of propolis and its pharmacological activities, including its mode of action against the kinetoplastids.

## 2. The Chemistry of Propolis

Bees use propolis to smoothen the inner walls of their hives or as gum to seal holes or cracks in the hives to keep intruders away. Propolis is also thought to protect the hive from bacterial, viral, and fungal infections [25,26]. Propolis color depends on its age and botanical source, and it could be yellow, green, red, dark brown, or transparent [27]. Both honeybees (*Apis mellifera* L.) and stingless bees (*Tetragonisca angustula* Illiger) produce propolis.

Bees collect plant resins and sticky exudates from flora in the area around the hive, usually from cracks in the bark or from leaf buds [28]. Then, they add salivary enzymes to the resins and mix it with beeswax, thereby forming propolis. Thus, the chemical composition of propolis varies with the geographical region, season, surrounding flora, and bee species [13,14,27,29,30,31,32]. Over 500 chemical compounds have been identified from various samples of propolis from different regions and seasons [27,33].

### 2.1. General Composition

The compounds most commonly isolated and identified from propolis are polyphenols, representing a diverse class of compounds. They include simple flavonoids, phenyl propanoids, phenols, benzoquinones, phenolic acids, acetophenones, phenylacetic acids, hydroxycinnamic acids, phenylpropenes, coumarins and isocoumarins, chromones, naphtoquinones, xanthones, stilbenes, anthraquinones, flavonoids, lignans, neolignans, lignins, and condensed tannins [27]. Although there are several reports on the chemical composition of propolis [27,34,35,36] many of these reports are based on hyphenated techniques such as GC–MS, HPLC–MS, and LC–MS/MS for the identification of constituents. Since these are dereplication techniques, this review is on compounds reportedly isolated and characterized using NMR and MS techniques in the period 2015–2020.

#### 2.1.1. Flavonoids

**Figure 1:**
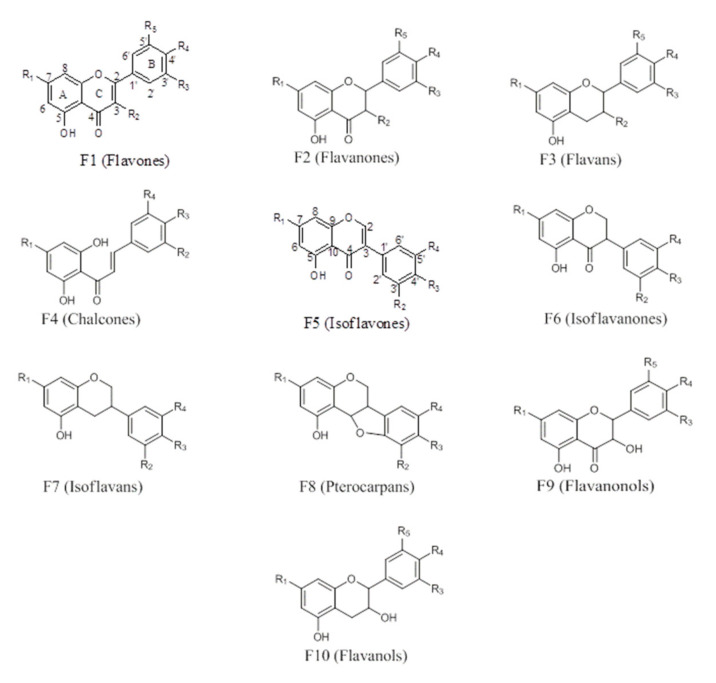
Flavonoid moieties. R1, R2, R3, R4 = H, OH, OCH_3_, *O*-sugar, prenyl, isoprenyl, etc.

The majority of phenolic compounds isolated from propolis are flavonoids (Figure 1). The variation between the compounds is mostly in the degree of saturation (Structure F2) or unsaturation (Structure F1) of ring C, the absence of the carbonyl group at C-4 (Structure F3), or the opening of ring C (Structure F4). Those with an unsaturated ring C containing a ketone at C-4 are known as flavones, while those with saturated ring C and C-4 ketone are flavanones. An –OH substitution at C-3 leads to flavanols (with an unsaturated ring C; F10) or flavanonols (with a saturated ring C; F9); compounds with the open C ring (F4) are chalcones. The compounds usually have a 5-OH substitution, and ring B can either be unsubstituted or substituted with –OH, –OCH_3_, or other substituents such as isoprenyl or sugars. These substituents could be at C-3, C-6, C-8, C-6′, or C-2′, and the –OH at position C-5 could be absent. Substitution of ring B to position C-3 instead of C2 produces the isoflavones (F5), isoflavanones (F6), and other moieties such as isoflavans (Structure F7) and pterocarpans (Structure F8). Many flavonoids have been identified in propolis; recent examples include isosativan, (2′-hydroxy-7,4′-dimethoxyisoflavan), liquiritigenin (**45**), isoliquiritigenin, formononetin (**46**), vestitol (**21**), neovestitol, medicarpin, 7-*O*-neovestitol, pinobanksin (**47**), pinocembrin, chrysin, and pinobanksin-3-*O*-acetate, astrapterocarpan, 3,8-dihydroxy-9-methoxy-pterocarpan, broussonin B, 8-prenylnaringenin (**20**), and gerontoxanthone H (**12**) [37,38,39].

#### 2.1.2. Phenyl Propanoids

**Figure 2:**
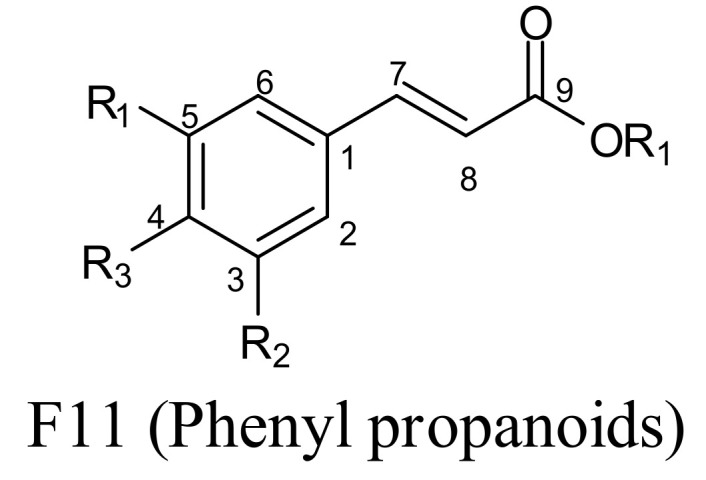
Phenyl propanoids. R_1_, R_2_, R_3_, R_4_ = H, OH, OCH_3_, *O*-sugar, prenyl, isoprenyl, phenethyl, benzyl, etc.

The next set of abundant compounds in propolis are phenyl propanoids (Structure F11; Figure 2). Here, the aromatic rings can also be substituted with –OH or –OCH_3_, and there could be phenethyl or benzyl substituents. Drupanin, 2,2-dimethylchromene-6-propenoic acid, artepillin C, baccharin, 7-methoxy-3-hydroxy-2,2-dimethyl-8-prenylchromane-6-propenoic acid, 2,2-dimethyl-8-hexylchromene-6-propenoic acid, and 3-hydroxy-2,2-dimethyl-8-prenylchromane-6-propenoic acid [40] are some examples of phenyl propanoids recently reported from propolis.

#### 2.1.3. Other Constituents

Other compounds that have been identified in propolis include aliphatic hydrocarbons, stilbenes, diterpenes, triterpenes [41], benzoic acid and its derivatives, benzaldehyde derivatives, cinnamyl alcohol, cinnamic acid and its derivatives, nicotinic acid, pantothenic acid, amino acids, carbohydrates, vitamins, and enzymes (glucose-6-phosphatase, acid phosphatase, adenosine triphosphatase and succinic dehydrogenase) [13]. Dereplication studies using HPLC–DAD–ESI-MS/MS identified the presence of pyrrolizidine alkaloids 7-(3-methoxy-2-methylbutyryl)-9-echimidinylretronecine and caffeoylquinic acid-*O*-arabinoside [42], but studies that report the isolation and identification of alkaloids from propolis using MS and NMR are rare. Trace elements such as Al, Ca, Fe, K, Mg, P, Zn, Cr, Ni, and Cu and possible toxic metals (As, Cd, and Pb) have also been reported [43,44,45].

### 2.2. Composition Based on Geographical Origin

Since the vegetation of different geographical regions varies, in addition to variations within the same region, and the phytochemicals in plants vary from season to season, the chemical composition of a propolis sample is determined primarily by its botanical source, the season, and the collection preferences of the bee species [13,14,29,30]. Thus, according to its botanical source, propolis may be classified into various chemotypes [30,46,47]; however, the assigned classifications unfortunately vary among authors as more types of propolis of different plant origins are being identified and characterized. However, the poplar type and Brazilian green propolis are the most widely available commercially and widely studied because of their medicinal properties. Poplar-type propolis is predominantly found in temperate regions and has been found to contain poplar bud phenolics [48]. Plant resins from the genus *Populus* (poplars) are the principal source of “poplar-type” propolis [49], found in parts of Europe, North America, New Zealand, temperate regions of Asia, and some regions of China. They typically contain aromatic (phenolic) acids and their esters, flavonoids, chalcones, dihydrochalcones, terpenoids, acyclic hydrocarbons, esters, alcohols, aldehydes, amino acids, aromatic hydrocarbons, fatty acids, ketones, sterols, sugars, and alcohols [50,51].

There is no clear geographic delineation for the classification of propolis from tropical regions such as Africa [14], because of the diversity of the tropical flora. Bees collecting propolis in tropical regions have a wider variety of plant sources; hence, there is little uniformity in the botanical source and, consequently, the phytochemicals. The compounds reported from tropical propolis include diterpenes, lignans, prenylated derivatives of *p*-coumaric acid, acetophenone, caffeic acid phenethyl ester, terpenoids, stilbenes, benzophenones, phenolic lipids, flavonoids, and diterpenic acids [13,27,30,38,52].

Over 148 compounds have been isolated from propolis from Africa (Table 1), South America (Table 2), Asia (Table 3), and Australia (Oceania) (Table 4) from different chemical classes. The majority of the compounds are flavonoids and their prenylated derivatives, whose isolation has been reported from propolis originating from every continent (except Antarctica). Flavonoids are abundant in the leaves, flowers, and fruits of the plants [53,54] from which bees collect resins and sticky exudates to make their propolis.

Triterpenoids are widely distributed in African propolis [15,16,55,57,58] but have also been reported in Bolivia [62,63], Brazil [61], Indonesia [28], and Thailand [67]. The most common triterpenes isolated from propolis are cycloartanes [15,28,58,61,62,63].

Other phytochemicals isolated from propolis within the period under review were not as widely distributed across all regions as the flavonoids and triterpenoids. However, studies indicate surprising similarity between propolis from Nigeria, Thailand, and Brazil, countries that could hardly be further apart, in their composition of triterpenoids, xanthones, and their prenylated derivatives [14,15,61,67]. Isolation of diterpenes is reported most from mainland Australia and the islands [71,72]. Australian propolis is unique, in that it contains stilbenes [73,74], which have not been reported in propolis from any other region. None of the studies reviewed reported the isolation of alkaloids from propolis. This could be due to the fact that alkaloids are much more abundant in roots and, since bees collect plant resins and sticky exudates from cracks in the bark or leaf buds of plants to make propolis, it is unlikely that alkaloids would be present in propolis.

Some of the compounds isolated from propolis are reported to have shown medicinal properties. For example, some flavonoids isolated from Nigerian propolis (astrapterocarpan, 3,8-dihydroxy-9-methoxy-pterocarpan, vesticarpan, medicarpin, vestitol, broussonin B, and 8-prenylnaringenin (**20**)) [39] or Bolivian propolis (3-prenyl-*p*-coumaric acid, kaempferol 3-methyl ether, and kaempferol 7-*O*-methyl ether) [63], were reported to have antioxidant properties.

## 3. Evidence for Propolis Protection against Bee Infections

Propolis is widely believed to be an important part of the bees’ defenses against infection of themselves and of their hive. The composition of propolis is principally dependent on the vegetation in the vicinity of the hive and on the bee species. Although the observation that some bee species collect only a small quantity of propolis leaves a question mark on the absolute requirement of propolis for bees, the notion that it protects bees from infection is backed up by a growing body of literature [76,77,78,79,80].

There is evidence showing a strong positive correlation between the amounts of propolis collected by bees and their heath condition, including their ability to produce viable broods (Simone-Finstrom and Spivak, 2010). Bees that collected larger quantities of propolis were reported to be healthier, producing viable broods and displaying superior hygienic behavior compared with the ones that collected less [76,77,81]. It was also found that bees usually respond to pathogens by collecting more propolis to ward off infections, while the immunity of the colony against infection is improved by the propolis envelop [78,82,83]. In addition, the microbiome of the bee colony is stabilized by propolis [84]. Colonies respond to *Ascophaera apis* (chalkbrood) by increasing resin collection for propolis, with hives with more propolis decreasing infection intensity [82]. However, the success of such a response depends on the type of vegetation in the vicinity of the hive and the chemical composition of its exudates.

It was shown that the ethanolic extracts of propolis were highly effective against *Paenibacillus larvae*, both in vitro and when field-tested in hives. It was not toxic to the bees when mixed with sugar syrup (oral administration), showing that propolis and its constituents are not toxic to bees. Propolis from Brazil also displayed significantly superior effects against *P. larvae* than propolis from Minnesota, United States of America (USA) [85,86], confirming that the activity of propolis depends on the vegetation around the hive.

Individual components isolated from propolis are also active against bee pathogens. Flavonoids and caffeates isolated from propolis displayed anti-*P. larvae* activity in vitro [87]. Another indication that certain chemical constituents in propolis offer an increased protective effect against *P. larvae* is that propolis from colonies free of *P. larvae* was reported to contain significantly more ferulic acid and coniferyl benzoate than propolis from colonies infected by this pathogen [88].

The protective efficacy of propolis for bees infested with *Varroa destructor* mites, a common pest of beehives, was recently directly confirmed. For instance, Argentinian propolis was found to be very effective against *Varroa* [89]. Furthermore, ethanolic extracts of German propolis were highly toxic to *Varroa destructor*, with a 10% *w/v* solution being lethal at 5 s contact [90], and Pusceddu et al. observed that raw propolis highly significantly increased the lifespan of *Varroa*-infected bees, almost completely reversing *Varroa*-associated mortality [91]. Moreover, the addition of natural propolis to hives reduced the titer of *Varroa*-transmitted deformed wing virus (DWV) [92] and *Varroa*-infected colonies specifically increased resin foraging [93]. It is not yet clear which chemical agents in propolis reduce the impact of *Varroa* infestation, but it was reported that the total polyphenolic content of propolis correlated with levels of *Varroa* infection in experimental hives in Sardinia [91]. Caffeic acid and pentenyl caffeates were found to be more abundant in propolis from *Varroa*-susceptible colonies [94], but this association requires further investigation in order to be confirmed as causal.

Propolis is also effective against bee infections caused by fungi. For instance, it was recently found that propolis fed to bees led to a significant reduction in *Nosema ceranae* infection [95]. Some acyl esters of flavonoids recently purified from propolis were characterized against two other honeybee pathogens: the fungus *Ascosphaera apis*, the causative agent of chalkbrood disease, and *Paenibacillus larvae* bacteria, which cause the disease American foulbrood. Pinobanksin 3-butyrate was identified as the most active chemical constituent against *A. apis*, while pinobanksin 3-octanoate was the most active agent against *P. larvae* [80].

It is becoming increasingly clear that the collection of propolis with strong antiprotozoal agents by bees is done purposefully. For instance, the microbiome of Scottish honeybees was reported to possess a high level of *Lotmaria passim* genetic material [96]. *L. passim* and *Crithidia mellificae* are trypanosomatids that are widespread in bee populations and are linked to colony losses that presently constitute a significant threat to honeybees; [96,97,98,99] characterized *C. mellificae,* together with *L. passim*, isolated from the honeybee *Apis mellifera*. However, the degree of pathogenicity of these trypanosomatid infections remains unclear but there is growing evidence for this. For instance, Gómez-Moracho et al. recently provided direct experimental evidence of the detrimental effects of the two trypanosomatids *L. passim* and *Crithidia mellificae* on honeybees in which honeybees inoculated with either *L. passim* or *C. mellificae* died faster than control bees [100]

Protozoal infections are spread within bee colonies through feces [101]. Therefore, bees may deliberately collect propolis that is active against trypanosomatids which they use for coating the surfaces within the hive to prevent disease transmission. It is, therefore, perhaps not surprising that propolis extract or purified chemical constituents are active against other trypanosomatids or kinetoplastids.

## 4. Propolis as an Anti-Kinetoplastid Agent

Currently there is a great interest in natural products-based drug discovery as a viable strategy for the treatment of diseases caused by the kinetoplastids. Among the most promising sources for such materials, propolis is being actively investigated based on its strong antiprotozoal activity [13,14]. Different types of propolis collected from diverse geographical locations all over the world have been attributed distinct pharmacological activities with promising results against various parasites belonging to the order kinetoplastida: *Trypanosoma* spp., *Leishmania* spp., and *Crithidia fasciculata,* a kinetoplastid model organism that is a close relative of *C. mellificae* (a bee pathogen) [102].

### 4.1. Antitrypanosomal Activity of Propolis

Several published papers have described the activity of propolis extracts and isolated components against a number of protozoan parasites. One of such reported biological properties, notwithstanding the quite distinct origins and compositions, is its in vitro and in vivo antitrypanosomal activity, reported by several authors [15,16,26,59,103,104,105,106,107,108,109,110,111,112].

#### 4.1.1. Identification of Bioactive Antitrypanosomal Compounds in Propolis Extracts

**Figure 3:**
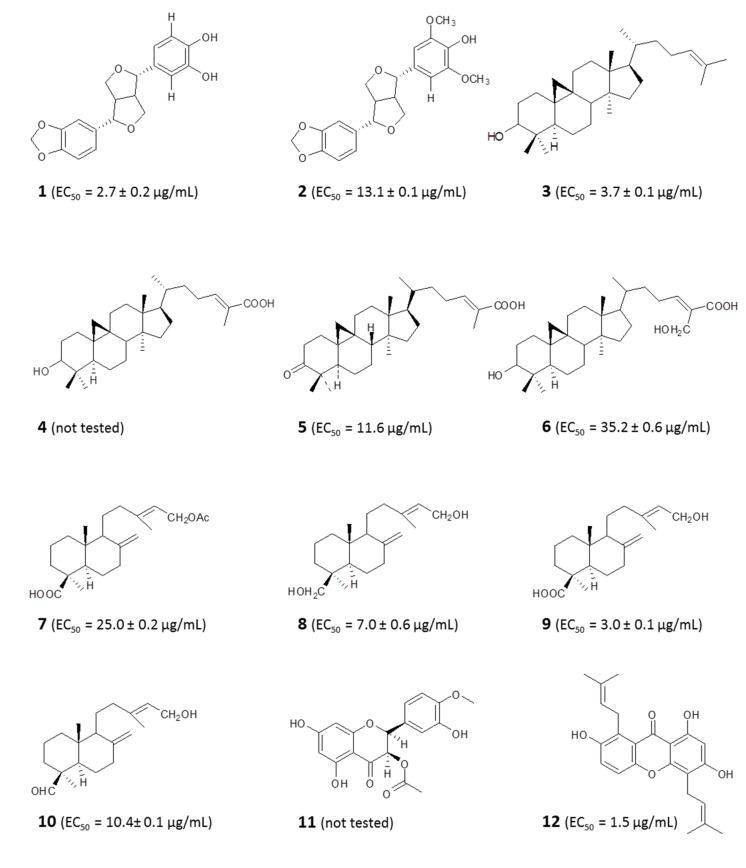

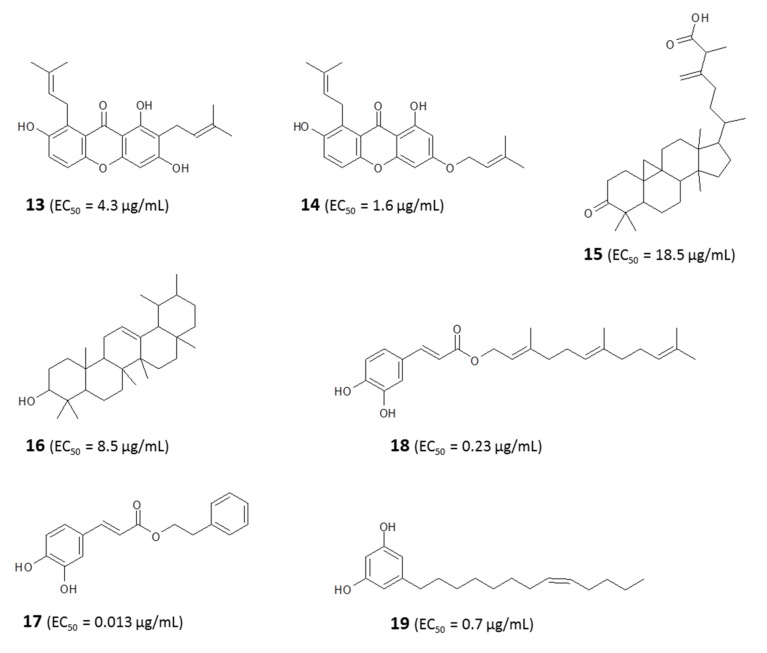
Structures of some compounds with activity against *Trypanosoma brucei*, isolated from propolis. The 50% effective concentrations (EC_50_) against the parasites in culture are indicated where available. Reference for EC_50_ values: Omar et al., 2016, 2017; Siheri et al., 2019 [14,15,59].

Libyan propolis has yielded lignans (demethylpiperitol (**1**) and 5′-methoxypiperitol (**2**)), cycloartane triterpenes (cycloartenol (**3**), mangeferolic acid, (**4**) mangiferonic acid (**5**), and 27-hydroxymangiferonic acid (**6**)), diterpenes (acetylisocupressic acid (**7**), agathadiol (**8**), isocupressic acid (**9**), and isoagatholal (**10**)), and a flavanone (taxifolin-3-acetyl-4′methyl ether (**11**)) with various levels of antitrypanosomal activity [59]. Studies of propolis samples from Nigeria also reported xanthones (Gerontoxanthone H (**12**), 6-deoxy-γ-mangostin (**13**), 1,7-dihydro-3-*O*-(3-methylbut-2-enyl)-8(3-methylbut-2-enyl) xanthone) (**14**), triterpenes (mangiferonic acid, ambonic acid (**15**), α-amyrin (**16**), and isoflavonoids with activity against *Trypanosoma brucei brucei* [14,15]. Two caffeic acid derivatives (β-phenethyl caffeate (**17**) and 2,2-dimethylallyl caffeate (**18**)) isolated from Fijian propolis were earlier reported to also have anti-*Trypanosoma* activity [18] (for structures, see Figure 3). Dereplication studies of European and Brazilian propolis samples associated their activity against *Trypanosoma* spp. with butyl and propionyl esters of pinobanksin, derivatives of benzopyran, caffeic acid, cinnamic acid, and flavonoids, where structures were not characterized by NMR [32,112].

#### 4.1.2. Metabolomic Profiling Revealed a Possible Mode of Action of Propolis In Vitro

We recently reported the activities of Libyan propolis against *T. b. brucei* and the isolation of an alkyl resorcinol from the extract. A fraction containing a cardol identified as bilobol (**19**) (Figure 3) exhibited a strong antitrypanosomal activity (50% effective concentration (EC_50_) = 0.7 μg/mL) and had no significant effect on a human cell line (human foreskin fibroblasts (HFF)), demonstrating excellent selectivity. Metabolomic profiling revealed the mechanism of action of the cardol-rich fraction. We observed a significant disturbance in the metabolism of choline phospholipids [59]. This suggests that (this component of) Libyan propolis might be targeting the cell membrane of trypanosomes, acting selectively on one class of phospholipids, rather like a surfactant, extracting lipid from the cell membrane, resulting in the leakage of high-energy phosphates. This mode of action of the cardol-rich fraction of Libyan propolis might be comparable to that of miltefosine, a well-established antileishmanial drug reported for selectively perturbing microbial membrane fluidity [113]. There are also reports suggesting that alkyl resorcinols disrupt cellular membrane phospholipid metabolism by inhibiting phospholipase C1 [114,115].

#### 4.1.3. Propolis Is Active against Drug-Sensitive and -Resistant Strains of *T. brucei*

Propolis is active against trypanosome various *Trypanosoma* strains including those that are highly resistant to current first-line drugs, as shown by our previous studies [14,15,112]. We determined the activities of compounds isolated from the ethanolic extracts of propolis collected from two regions in Nigeria against a panel of *T. brucei* strains including (i) *T. brucei* Lister 427 wild type (WT), which is the standard drug-sensitive control; (ii) an aquaglyceroporin2/3-null (AQP2/3-KO) strain, from which the TbAQP2/AQP3 locus was deleted [116], coding for the critical drug transporter HAPT1 [117,118] and, consequently, resistant to pentamidine and melarsoprol [119]; (iii) a multidrug-resistant strain, B48, adapted from Lister 427WT by deletion of the TbAT1/P2 drug transporter [120] and subsequent adaptation to very high concentrations of pentamidine in vitro [121], making the strain highly resistant to all diamidine- and melaminophenyl arsenical-based drugs.

**Figure 4:**
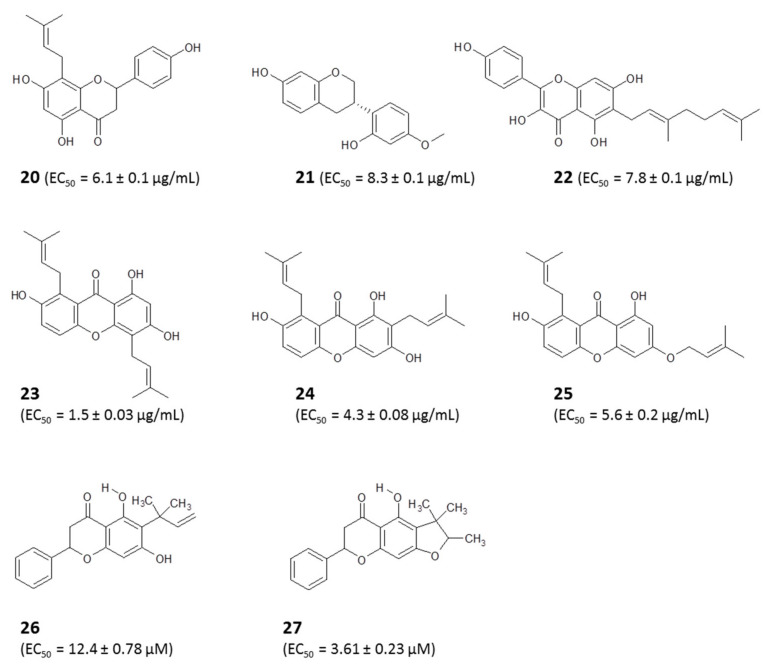
Antitrypanosomal compounds from Nigerian propolis. EC_50_ values are from Omar et al., 2016, 2017 [14,15].

The crude extracts all contained complex mixtures of natural compounds, of which 8-prenylnaringenin (**20**) was the most active of the purified compounds at 6.1 ± 0.1 µg/mL, and vestitol (**21**) and macarangin (**22**) displayed similar activities. Importantly, none of the diamidine- and arsenical-resistant strains were co-resistant to either the crude ethanolic extracts or the isolated compounds [15] (for structures, see Figure 4). This significant finding suggests that propolis may be a potential solution to the present challenge of drug resistance facing chemotherapy of human [122] and veterinary [123] trypanosomiasis.

A profiling of 12 additional propolis samples, collected from eight regions in Nigeria, identified three xanthones, 1,3,7-trihydroxy-4,8-di-(3-methylbut-2-enyl) xanthone (**23**), 1,3,7-trihydroxy-2,8-di-(3-methylbut-2-enyl) xanthone (**24**), and a xanthone that was previously undescribed (1,7-dihydroxy-3-*O*-(3-methylbut-2-enyl),(3-methylbut-2-enyl)xanthone) (**25**) (Figure 4), as well as three triterpenes, mangiferonic acid (**5**), a mixture of α-amyrin (**16**) with mangiferonic acid (1:3), and ambonic acid (**15**). These compounds all displayed trypanocidal activities against wild-type and resistant strains of *T. b. brucei* with EC_50_ values below 25 µg/mL but only the xanthones displayed high activity, i.e., EC_50_ values <5 μg/mL; xanthone (**23**) was the most active, with an EC_50_ of 1.5 ± 0.03 μg/mL. Interestingly, the compound displayed even higher activity against the AQP2 knockout strain (0.8 ± 0.02 µg/mL, *p* < 0.001), which was >30-fold resistant to pentamidine [14]. Similarly, and very recently, a bioassay-guided fractionation of Tanzanian and Zambian propolis samples led to the isolation of two novel flavanones with antitrypanosomal activities. The compounds were identified as 6-(1,1-dimethylallyl)pinocembrin (**26**) from the Zambian propolis sample and 5-hydroxy-4″,4″-dimethyl-5″-methyl-5″-*H*-dihydrofurano [2″,3″,6,7]flavanone (**27**) obtained from the Tanzanian propolis sample [124].

#### 4.1.4. Propolis Contains Antitrypanosomal Activities Regardless of Geographical Location

Propolis samples collected from different locations within a country may possess different antitrypanosomal efficacies, due to differences in vegetation and/or bee species. For instance, ethanolic extracts obtained from 12 propolis samples collected from various regions in Libya showed a wide range of activity against *T. brucei* (EC_50_ value 1.67 μg/mL–39.38 μg/mL) [16]. Similarly, the antitrypanosomal activity of 35 propolis samples collected from different parts of Europe displayed varying activities against wild-type (WT) *T. brucei* and *T. congolense*, including the multidrug resistant strain *T. brucei* B48. Four of these samples showed high activity, while 23 had an intermediate activity (5–10 μg/mL) against WT and B48 *T. brucei* [112]. For the purpose of comparative analysis, *C. fasciculata* was also included and tested in parallel with these *Trypanosoma* species.

Interestingly, there was a very good overall correlation between the activities of each of the samples against the various kinetoplastid species, particularly between the *Trypanosoma* species/strains. This is very important because “African” trypanosomiasis is caused by multiple *Trypanosoma* species including *T. congolense*, *T. vivax*, *T. b. brucei*, *T. b. gambiense*, and *T. b. rhodesiense*; moreover, African trypanosomes that have adapted to non-tsetse fly transmission, including *T. evansi*, *T. equiperdum*, and *T. vivax*, have spread far beyond the African continent [123,125]. Few if any current drugs are effective against all these species. Another highly significant finding in the report was the very good correlation observed between the activity against drug-sensitive and drug-resistant strains, with the activities of the propolis samples against the highly resistant strain B48 on average performing even better than against the parental strains. This report, therefore, confirmed that cross-resistance with the current available trypanocidal drugs is less likely for propolis-derived compounds. Mechanistically, this is due to drug resistance in African trypanosomes being mostly linked to loss of drug transporters [118], which would not be the import mechanism for the structurally very different propolis-derived natural compounds.

#### 4.1.5. Propolis Is Active In Vitro and In Vivo Against Trypanosomes

**Figure 5:**
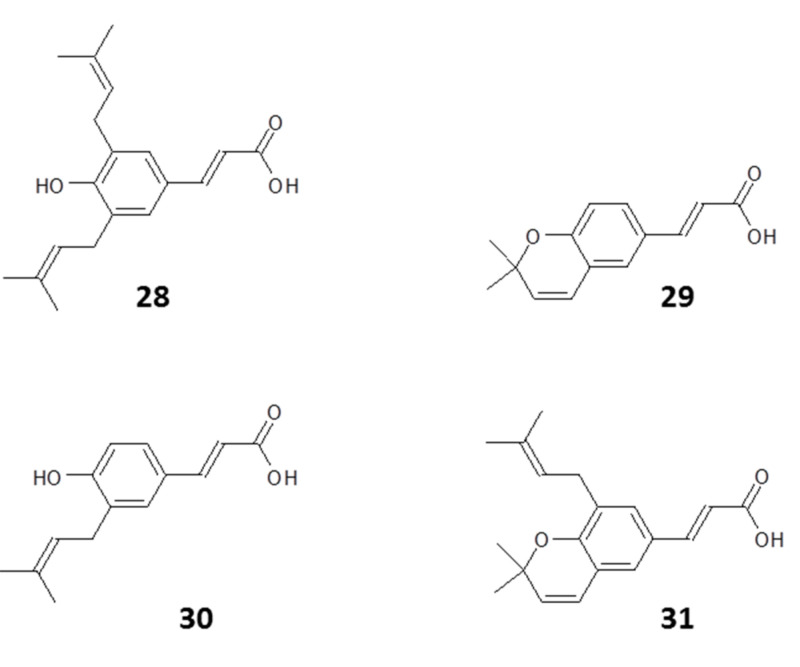
Structures of some phenolic compounds from Brazilian propolis active against *T. cruzi*. [105].

There are also several reports in the literature showing that propolis has in vitro activity against *T. cruzi*, the causative agent of American trypanosomiasis [126]. Marcucci et al. purified four bioactive phenolic compounds from Brazilian propolis: 3,5-diprenyl-4-hydroxycinnamic acid (**28**), 2,2-dimethyl-6-carboxyethenyl-8-prenyl-2*H*-1-benzopyran (**29**), 3-prenyl-4-hydroxycinnamic acid (**30**), and 2,2-dimethyl-6-carboxyethenyl-2*H*-1-benzopyran (**31**) (structures in Figure 5). All four phenolic compounds showed activity against *T. cruzi* Y strain trypomastigote (bloodstream) forms after 24 h exposure. The 24 h EC_50_ values of 0.72–2.64 mg/mL are high but reflect an incubation time of 24 h at 4 °C, chosen to test the feasibility of decontaminating donated blood batches in a blood bank [105]. Dantas et al. also observed anti-*T. cruzi* activity when assessing the in vitro antitrypanosomal effects of ethanolic and supercritical extracts of green, brown, and red, propolis from different regions of Brazil against Y strain epimastigote (insect) forms, albeit at high concentrations. Brazilian red propolis appeared to be the most active in these tests [127].

Extracts of Brazilian green propolis were used as oral treatment for acute infections of *T. cruzi* in mice (25–300 mg/kg body weight/day for 10 days). The mice showed reduced parasitemia and increased survival with no observable toxicity [128]. Similarly, rats infected with *T. b. brucei* and treated orally with Nigerian red propolis extracts (600 and 400 mg/kg for 5 days) had significantly reduced parasitemia, with higher red cell counts, packed-cell volume, and weight gain than untreated (control) mice [129]. It is important to note that these therapeutic effects were achieved with the crude extracts rather than purified active compounds, which would be expected to have stronger beneficial effects.

Chagas disease features *T. cruzi* parasites in the bloodstream (trypomastigotes) and inside mammalian host cells (amastigotes) and, crucially, propolis is active against both life-cycle forms. Treatment of heart muscle cells and macrophages infected with amastigotes with the ethanolic propolis extracts dose- and time-dependently reduced parasite loads, and 100 μg/mL of the extract fully lysed trypomastigotes within 24 h [103].

#### 4.1.6. Direct Antiparasitic Efficacy and Sites of Action of Propolis in *T. cruzi*

One of the cellular sites of propolis action in *T. cruzi* is the mitochondrion. Treatment of *T. cruzi*-infected skeletal muscle cells with the ethanolic fraction of a Bulgarian propolis caused a decrease in the proliferation of intracellular amastigotes, swelling of the parasite’s mitochondrion, and concentric membrane structures appearing in the mitochondrial matrix. It also inflicted ultrastructural changes in the mitochondrion–kinetoplast complex of trypomastigotes and in the reservosomes of epimastigotes, characterized by distinct changes in their electron density and morphology. Reservosomes are large membrane-bound organelles located at the posterior end of the epimastigotes of *T. cruzi*, but absent in trypomastigote and amastigote forms [130]. The presence of electrolucent rod-shaped inclusions was also observed [106].

Most studies report that amastigotes derived from cell culture were more susceptible to treatment with ethanolic propolis fractions than trypomastigotes [131] and epimastigotes [132], and it may be that different compounds in these complex extracts are active on the diverse forms. Investigations by [128] to determine the cellular target of the ethanolic extract of Brazilian green propolis on various life-cycle stages of *T. cruzi* found different effects on epimastigotes (alterations in the ultrastructure of the mitochondrion, reservosomes, and Golgi complex) and trypomastigotes (loss of integrity and functionality of plasma membrane) [128].

#### 4.1.7. Indirect Antiparasite Efficacy of Propolis via Immune Modulation in *T. cruzi* Infection

Apart from the direct antiparasite efficacy, propolis also interferes with the basic functions of the immune cells. Orally administered ethanolic extracts (50 mg/kg body weight) of Bulgarian propolis to *T. cruzi*-infected mice decreased parasitemia with no observable hepatic or renal damage. The treatment also decreased the spleen mass, including modulation of inflammatory reactions such as preferential expansion of CD8^+^ cells [107].

This immunomodulatory mechanism of action is likely associated with an increased resistance to infection, because activated CD4^+^ cells are known to increase the production of cytokines including IL-2 and IFN-γ, which are associated with differentiation and activation of the CD8^+^ T cells, resulting in an increased immune response.

### 4.2. Anti-Leishmania Effects of Propolis

Leishmaniasis affects over 12 million people and is endemic in 88 countries across the tropics and subtropical regions of the world. Like trypanosomiasis, almost all chemotherapeutic options for leishmaniasis have unacceptable side effects, and there are as yet no vaccines for human use. There is currently an intense search for alternative safe anti-*Leishmania* chemotherapy from propolis. Several studies have shown that propolis obtained from diverse origins possesses antileishmanial activity due to the presence of flavonoids [16,17,18,19,20,21,22,23,24].

#### 4.2.1. Identification of Active Antileishmanial Compounds in Propolis Extracts

**Figure 6:**
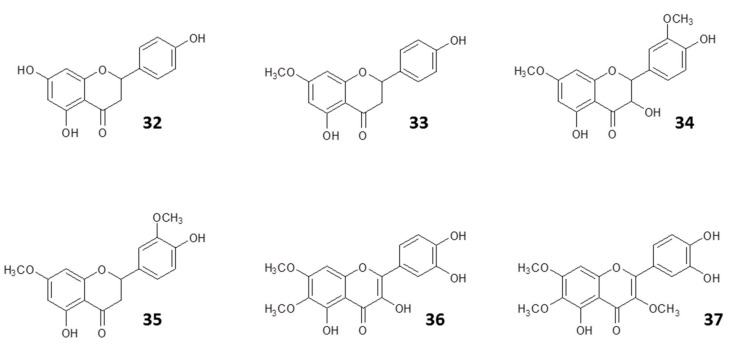
Structures of some flavonoids identified in the active fraction of propolis from Ecuador with antileishmanial activity [63].

In fact, Ecuadorian propolis high in flavonoids including (5,7,4′-trihydroxyflavanone (**32**), 5,4′-dihydroxy-7-methoxyflavanone (**33**), 3,5,4′-trihydroxy-7,3′-dimethoxyflavanone (**34**), 5,4′-dihydroxy-7,3′-dimethoxyflavanone (**35**), 3,5,3′,4′-tetrahydroxy-6,7-dimethoxy flavone (**36**), and 3,5,4′-trihydroxy-7,3′-dimethoxy flavone (**37**)) was found to possess much better antileishmanial activity than Ecuadorian propolis samples rich in triterpenic alcohols and acetyl triterpenes and inhibited the growth of *L. amazonensis* amastigotes and promastigotes [63]; structures are shown in Figure 6.

**Figure 7:**
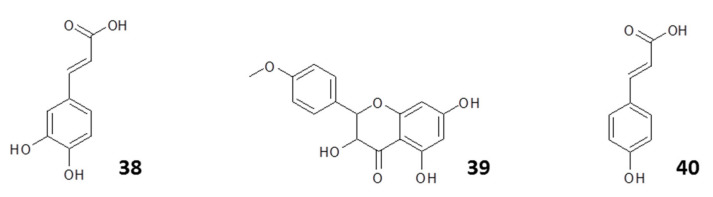
Structures of some compounds isolated from propolis with synergetic activity against *Leishmania (Viannia) braziliensis* [23].

One of the common factors usually evaluated when investigating the pharmacological activities of propolis is isolating the active component and testing it against a pathogen. However, studies have shown that a synergistic effect offered by several chemical constituents of the mixture may exist and should not be ignored when evaluating its biological activities. In some experiments to study the effect of propolis extracts on *Leishmania,* the active principles that contributed to the inhibition of the proliferation of the promastigote forms of *Leishmania (viannia) braziliensis* were caffeic acid (**38**), aromadendrine-4′-methyl ether (dihydrokaemferide) (**39**), *p*-coumaric acid (**40**), 3,5-diprenyl-*p*-coumaric (artepillin C) (**28**), and 3-prenyl-*p*-coumaric (**30**) acid (Figure 7). These compounds together reduced the lesions caused by the infection [23]. Importantly, extracts of Bulgarian propolis, rich in flavonoids, displayed activity against several old- and new-world *Leishmania* species, *L. amazonensis*, *L. braziliensis, L. chagasi*, and *L. major* [24], indicating a broad spectrum of antileishmanial activity. This is considered to be essential for the development of antileishmanial drugs for the international market, although it could be envisaged that propolis-derived treatments could be developed to fill a more local need.

Similarly, Nina et al. [65] assayed Bolivian propolis extracts and their active compound against promastigotes of *L. braziliensis* and *L. amazonensis*. They found that propolis rich in phenolic compounds displayed superior antibacterial and antileishmanial activity than those containing mostly triterpenes. The methanol extracts showed leishmanicidal activity against promastigotes of both species with MIC_100_ values in a tight range of 7.8 to 12.1 μg/mL depending on the *Leishmania* species and the geographical origin of the propolis, further reinforcing the activity of propolis against multiple *Leishmania* species.

It thus appears that, as for trypanocidal activity, propolis from different countries are also active against *Leishmania*, despite a great variation in chemical constituents, although the level of antileishmanial activity does depend on the propolis constitution. Extracts of 35 propolis samples collected from different parts of Europe were assessed for antileishmanial activity against wild-type and miltefosine-APC12-resistant strains (C12R×, resistance factor (RF) >600-fold) of *L. mexicana* promastigotes. All the samples showed a high or moderate level of activity against the wild-type strain (EC_50_ 0.35–5.67 μg/mL) and the miltefosine-APC12-resistant strain (0.28–1.55 μg/mL); the best activity was again noticed in propolis from Bulgaria [112]. Interestingly, in most cases, the propolis samples were more active against the resistant strain (RF was as low as 0.23, i.e., >4-fold more sensitive). Although very preliminary, this study offers the suggestion that propolis could offer a solution to the current issue of drug resistance in *Leishmania* chemotherapy, at least for miltefosine. Clearly, much more research into this important possibility is required, including the mechanism by which the resistant strains would become particularly sensitized to (which?) specific constituents of European propolis.

#### 4.2.2. Effects of Propolis on Infected Macrophages

Propolis is capable of killing *Leishmania* in macrophages, thereby reducing parasitemia load. For instance, Santana et al. reported effects of brown propolis from the semiarid region of Piauí, Brazil, against both promastigotes and intramacrophage amastigotes, with the dichloromethane fraction being the most active [133]. A survey of Cuban propolis samples particularly highlighted the activity of a yellow propolis rich in acetyl triterpenes against intramacrophage *L. infantum*, as well as against *T. cruzi* and *T. brucei* [134]. However, that study also found that the selectivity of the yellow propolis samples over MRC-5 human fibroblasts was quite low. In contrast, [135] found that ethanolic extracts of green and red propolis against *L. braziliensis* promastigote-infected BALB/c mouse-derived macrophages reduced the *L. braziliensis* load without observable toxicity to the macrophages. Nonetheless, the red propolis showed a stronger parasite reduction than the green propolis extract and, at a concentration of 100 μg/mL, showed almost the same effect as the standard drug amphotericin B. Ethanolic extracts of Brazilian red propolis were also active against promastigotes and extracellular amastigote forms of *L. amazonensis* in infected macrophages in a time- and dose-dependent manner, with low toxicity to noninfected macrophage controls [136]. This extract was previously reported to be rich in benzophenones and prenylated compounds [105,137].

#### 4.2.3. Propolis in Animal Models of Leishmaniasis

The above studies all describe the antileishmanial activity of propolis in vitro and sometimes only on the promastigote (insect) form. However, a very recent study showed that propolis was as active as one of the available standard drugs when tested against cutaneous leishmaniasis in an in vivo model of *L. major* infection. Tavakoli et al. observed that the ethanolic extract of Iranian propolis inhibited the growth of promastigote forms of *L. major*, as well as the standard drug Glucantime (meglumine antimoniate) at concentrations >37.5 μg/mL in vitro (*p* > 0.05). More importantly, in a mouse model of cutaneous leishmaniasis (*L. major*), treatment with 4% ethanolic propolis extract (4 g extract plus 96 g vaseline–oserin) reduced the size of skin lesions with similar efficacy as Glucantime, the standard antileishmanial treatment in much of the world [138].

Some of the in vivo benefits of propolis may be through direct action on the parasite. However, a water extract of green propolis was able to prevent the progression of *L. infantum*-induced lesions in the liver during infection, even better than some of the commercially available drugs such as Glucantime, by reducing the parasite-induced lesions and secondary chronic inflammatory processes in the liver [139]. Da Silva et al. similarly showed that propolis treatment reduced leishmaniasis-associated liver inflammation, reporting decreases in the levels of liver *N*-acetyl-β-glucosaminidase and myeloperoxidase activity and of proinflammatory cytokines, as well as lower collagen fiber deposition, and plasma aspartate [140]. In contrast, the levels of anti-inflammatory cytokine were increased, and hepatosplenomegaly was at least partially reversed [139,140]. Propolis also decreases the side effects of meglumine antimoniate in the host [139].

#### 4.2.4. Synergy of Propolis and Mainstream Antileishmanials

Propolis also exhibits synergistic leishmanicidal effect when combined with standard drugs such as Glucantime or amphotericin B. Ayres et al. reported that a gel prepared from Brazilian red propolis reduced the amount of exudate from leishmanial skin lesions, particularly when combined with Glucantime [141]. Very recently, Jihene et al. assessed the antileishmanial effect of an essential oil from Tunisian propolis and its combination with amphotericin B against clinical isolates of *L. infantum* and *L. major* [142]. The essential oil showed good activity against promastigote forms of *L. infantum* and *L. major* (EC_50_ = 5.29 μg/mL and 3.67 μg/mL, respectively) and against the amastigote forms (EC_50_ = 7.38 μg/mL and 4.96 μg/mL, respectively), with low cytotoxicity. The very similar activity against the promastigote and amastigote forms is important for the evaluation of other studies that tested only against the easy-to-culture promastigote stage. A synergistic efficacy was observed when the essential oil was combined with amphotericin B (fractional inhibitory concentration (FIC) = 0.37). The active principles were further identified as α-pinene (**41**) (36.7%), α-cedrol (**42**) (6.7%), totarol (**43**) (6.6%), and dehydroabietane (**44**) (5.2%). The authors attributed the antileishmanial efficacy of the essential oil mostly to α-pinene, which has a reported moderate activity against promastigotes and amastigotes [143], synergistically with that of the minor but more potent components, especially α-cedrol (EC_50_ = 1.5 µM) [142] and totarol (EC_50_ = 12.2 µM) to *L. donovani* promastigotes [144]. However, that putative synergism remains to be experimentally tested. The mechanism of action of the propolis essential oil was proposed to be activation of macrophages by hyperproduction of NO, and this could play a role, but would not explain the effects against promastigotes, which was in fact higher than against intramacrophage amastigotes.

#### 4.2.5. Direct Antiparasite and Indirect Effects of Propolis on Intramacrophage Amastigotes via Immunomodulation

Upregulation of the macrophage microbicidal activities is one of the reported modes of action of propolis in *Leishmania* infection, and it is becoming increasingly clear that immunomodulation is a major mechanism of action of propolis in *Leishmania* infection. Brazilian propolis extracts with high concentrations of phenolic compounds (flavonoids, benzopyrans, and aromatic acids), di- and triterpenes, and essential oils showed a direct inhibitory effect on promastigote forms of *L. braziliensis*, with a concentration of 100 µg/mL of propolis extract as effective as 250 µg/mL Glucantime. Interestingly, the preincubation of macrophages with just 5 µg/mL or 10 µg/mL propolis extract induced them to take up more promastigotes but resulted in a strong reduction in recovered promastigotes after 5 days [17], demonstrating an increased proficiency of the macrophages to kill the parasites internalized. The authors linked this observation to an observed increase in the level of TNF-α in mice pretreated with propolis extracts, coupled with the downregulation of IL-12 during the infection [17]. Orsatti et al. investigated immunomodulation in mice treated for 3 days with ethanolic extracts of propolis and reported an increase in the expression of Toll-like receptors (TLR)-2 and TLR-4 in macrophages, as well as an increase in the production of proinflammatory cytokines IL-1β and IL-6, indicators of activation of the innate immune response [143].

Moreover, dry, alcoholic, and glycolic propolis extracts at various concentrations (10, 50, or 100 µg/mL) showed, again, a dose-dependent effect on the viability of promastigotes of *L. braziliensis* in culture, as well as reduced parasite loads in macrophages. There were reduced levels of superoxide and nitric oxide in activated macrophages infected with *L. braziliensis*, as well as increased activity of superoxide dismutase (SOD), following treatment by the alcoholic and glycolic extracts; these are all antioxidant responses. However, the inflammatory profile of macrophages was significantly modified by the dry propolis extract via upmodulating TNF-α, while downmodulating the production of IL-10 and TGF-β [145], changes that lead to a greater activation of the cells.

These data put together suggest that propolis extracts or its constituents are well tolerated by macrophages and can increase the mechanisms of macrophage activation, resulting in the neutralization of *Leishmania*.

#### 4.2.6. Nanotechnology in Delivering Propolis Therapy

Several technological advances have been made with regard to the use of propolis in leishmaniasis drug discovery. Propolis has been loaded onto polymeric nanoparticles for targeted drug delivery [146,147]. Some of the most accepted drug delivery vehicles with pharmaceutical applications are polymeric nanoparticles and liposomes. This is due to the advantages of target delivery, nontoxicity, biocompatibility, biodegradability, controlled drug release, and stability during storage, all leading to an increased therapeutic efficacy [148].

**Figure 8:**
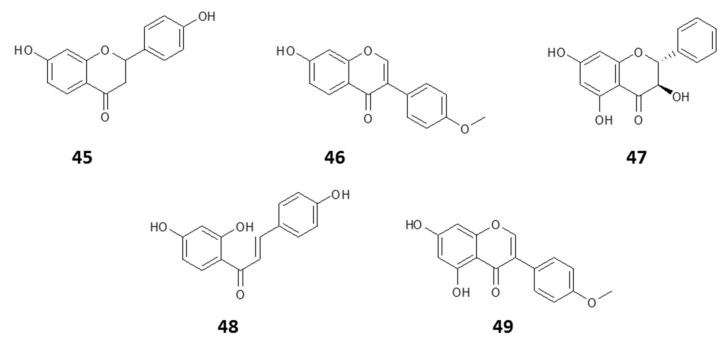
Structures of some flavonoids with antileishmanial activity isolated from Brazilian propolis and loaded into polymeric nanoparticles [149].

Correspondingly, [149] assessed the efficacy of polymeric nanoparticles loaded with an ethanolic extract of Brazilian red propolis for antileishmanial therapies in a multiple-constituent co-delivery system. Using a nanoprecipitation method, polymeric nanoparticles (poly-ε-caprolactone and pluronic) were loaded with red propolis extract and were characterized for leishmanicidal activity. The red propolis nanoparticles were stable without any aggregation phenomenon observed during a 1 month period, while exhibiting antileishmanial activity with an EC_50_ value of 31–47 μg/mL against *L. braziliensis* promastigotes in vitro. Analysis of the propolis extract identified several flavonoids as the potential active compounds, specifically liquiritigenin (**45**), formononetin (**46**), pinobanksin (**47**), isoliquiritigenin (**48**), and biochanin A (**49**) (structures in Figure 8).

In summary, it appears that the major antileishmanial chemical constituents of propolis are specific flavonoids and certain metabolites of caffeic acid [150,151], whereas the most reported mode of action of propolis against *Leishmania* is immunomodulation through the activation of macrophages, although it is clear that direct antileishmanial effects also importantly contribute given the in vitro observations.

### 4.3. Effects on Crithidia fasciculata

*Crithidia, Leishmania*, and *Trypanosoma* are members of the order Kinetoplastida, and *Crithidia fasciculata* is a very close relative of *C. mellificae*, a parasite of honeybees, and of *Crithidia bombi*, the bumble bee pathogen. *C. mellificae* has been reported to be significantly responsible for the winter mortality often observed in beehives across Western European [152]. Considering that these pathogens are closely related genetically and consequently possess comparable metabolism and life cycle, *Crithidia fasciculata* was adopted as an accessible and well-researched model organism for the study of these important bee infections.

**Figure 9:**
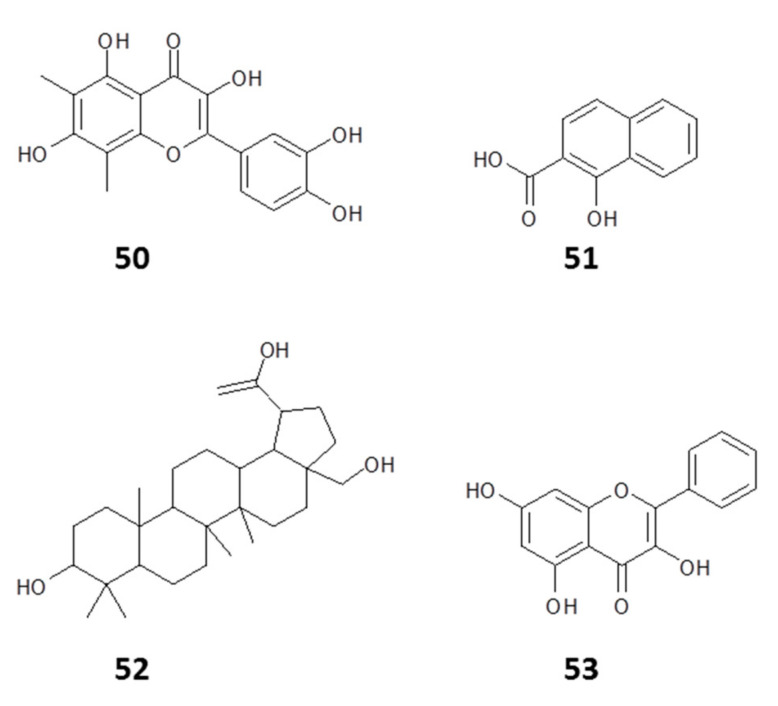
Compounds identified in the ethanolic extracts of Libyan propolis samples with anticrithidial activity [16,153].

Accordingly, our lab successfully developed strategies for the screening of propolis extracts and fractions on *C. fasciculata* [16] and used this approach to chemically characterize various propolis samples. The strong anti-kinetoplastid activity of propolis extracts seems to be a virtually constant feature in the literature reports and our own experience, which strongly indicates that bees deliberately collect propolis to protect themselves against invasion of their hive by pathogens, including *Crithidia* species, coating the hive in an antimicrobial substance. Investigation of the activity of propolis on *C. fasciculata* by screening of ethanolic extracts of 12 Libyan propolis samples showed that all the extracts were active but exhibited a range of EC_50_, with the most active extract having an EC_50_ of 6.5 μg/mL. This activity was correlated strongly with dimethylquercetin (**50**) and a derivative of hydroxynaphthoic acid (**51**) in an orthogonal partial least squares (OPLS) model of the anticrithidial activity [16] (structures in Figure 9).

Extracts of propolis from Papua New Guinea inhibited the growth of *C. fasciculata* [153]. Chemical profiling of the extract conducted using negative ion spray ESI (LC–MS) revealed a high concentration of triterpenes in the active (ethanolic) fraction, indicating that the observed activity was likely due to the inhibitory action of triterpenes on the viability of *C. fasciculata.* Nine compounds were subsequently purified from the ethanolic fraction and their structural elucidation revealed eight cycloartane-type triterpenes and a pentacyclic triterpene (20-hydroxybetulin (**52**, Figure 9)), which on further testing gave the best activity against *C. fasciculata* [153].

To assess the effect of geographical location of the propolis samples on the activity against *C. fasciculata* and, by extension, the possible effects on bee pathogens caused by the trypanosomatids, the anticrithidial activity of extracts from 35 propolis samples from different parts of Europe was investigated. Moderate-to-high levels of anticrithidial activity were observed for all 35 samples, with EC_50_ values in the range of 2.5–22.7 µg/mL. OPLS modeling of the chemical constituents correlated the highest activity with pinobanksin (**47**) and a methyl ether of galangin (**53**, Figure 9) [112].

A higher activity against *C. fasciculata* and *T. brucei* was observed in a comparative study of propolis efficacy against the kinetoplastids with ethanolic extracts of Nigerian propolis samples. The triterpenoids mangiferonic acid (**5**, EC_50_ = 11.6 μg/mL), ambonic acid (**15**, EC_50_ = 18.5 μg/mL), and α-amyrin (**16**, EC_50_ = 8.5 μg/mL), and the xanthones gerontoxanthone H (**12**, EC_50_ = 1.2 μg/mL), 6-deoxy-γ-mangostin (**13**, EC_50_ = 4.3 μg/mL) and 1,7-dihydro-3-*O*-(3-methylbut-2-enyl)-8-(3-methylbut-2-enyl) xanthone (**14**, EC_50_ = 1.6 μg/mL) were isolated from the samples. The crude extract samples were found to have higher antitrypanosomal activity than most of the isolated compounds. EC_50_ values of the most active crude samples were 1.2 and 4.2 µg/mL for *C. fasciculata* and *T. brucei*, respectively [14]. Other triterpenoids, diterpenes, lignans, flavonoids, etc. isolated from propolis samples collected from different geographical regions (Table 1) are reported to have different levels of anti-kinetoplastid activity. This shows that, regardless of geographical location, propolis contains anti-kinetoplastid compounds. Overall, the consistently observed activity against *C. fasciculata* gives support to the hypothesis that bees collect propolis specifically to protect them from infections caused by pathogens, particularly those caused by species of *Crithidia*, well-known bee pathogens that are quite closely related to the human pathogens *Leishmania* and *Trypanosoma* [102], and the closely related *L. passim* which has been found to be abundant in bees [98,99]. This view was further strengthened by the surprisingly excellent correlation observed between the EC_50_ values of Nigerian propolis fractions against *T. brucei* and *C. fasciculata* [14]. This, however, further strengthens the case for the development of drugs against other trypanosomatids such as *Leishmania* and *Trypanosoma* species from propolis samples.

## 5. Conclusions

Propolis samples possess a wide range of chemical constituents, which largely depend on the geographical location where it was collected, in addition to seasonal variations stemming from the vegetation in the locality. The compounds present in propolis, particularly the different types of flavonoids, appear to be responsible for the observed broad spectrum of biological activities, with some individual compounds in the samples showing activity against different organisms, particularly the kinetoplastids. The consistently observed high levels of antiprotozoal activity of propolis extracts, especially against the kinetoplastids, together with the recent findings of Regan et al. [96] regarding the presence of DNA of several protozoan parasite species in the bee metagenome, indicate that these pathogens may be exerting more pressure on the health of bee colonies than heretofore known. Therefore, there remains a lot to be understood regarding the role of propolis in bee health, but it now looks certain that the near-universal presence of anti-kinetoplastid activity in bee propolis is not incidental. Thus, propolis is a source of natural compounds, preselected by evolution, against important neglected diseases such as leishmaniasis, sleeping sickness, and Chagas disease. The broad anti-kinetoplastid activity of propolis components reviewed here, together with the generally low toxicity to macrophages and experimental animals, beneficial immunomodulation, and our recent findings that the main bioactive metabolites (flavonoids) present in propolis are well absorbed and tolerated by the human body [154], gives ample scope for further investigations toward the rational development of anti-kinetoplastid drugs that will replace the existing ones, which have many undesirable side effects and often suffer from drug resistance after decades of use [155]. However, questions remain with regard to the efficacy of propolis components (such as flavonoids) in vivo since, although these compounds are often well absorbed, they are also rapidly metabolized particularly to glucuronides and sulfates. This problem has been extensively addressed in previous papers and reviews, and the following points are of importance [156,157,158,159,160]:

(i) In some cases, the biological activity of flavonoids is not improved or sometimes increased by conjugation.
(ii) At higher doses and in samples containing a mixture of flavonoids, there may be incomplete conjugation of particular flavonoids.
(iii) It is possible that flavonoid metabolites can become deconjugated.

Given the generally low toxicity of propolis and the high toxicity of many of the existing antiprotozoal drugs, it may be possible to optimize the efficacy of propolis treatments by giving a high dosage. In addition, if crude extracts were to be used as treatment, it would be important to set a standard, perhaps on the basis of the concentrations of the key components in the extracts.

The mode of action of propolis depends on the organism it is acting on, and ranges from direct effects on growth and/or viability of the pathogen to immunomodulation via macrophage activation or cytokine changes, perturbation of the cell membrane architecture through phospholipid disturbances, and mitochondrial targets. Given the complexity and variability of propolis, mechanism-of-action studies are particularly fraught and, frankly, lagging. The mechanism of any activity of “propolis” could only be defined if “propolis” itself is perfectly defined and standardized. Although there has been excellent analytic work to identify the constituents of specific propolis samples, each of these have been very different. Logically, then, mechanistic studies can only be performed with individual compounds shown to be present and even dominant in some types of propolis. However, this potentially loses synergy (or, conversely, antagonism) between components in complex propolis samples: a well-understood conundrum for ethnopharmacologists and phytochemists. Meanwhile, identification of specific cellular targets makes limited sense until a genuine lead compound is chosen from among the large number of compounds that show promise. The criteria for the lead compound need to be agreed upon but must surely include high efficacy, low toxicity, metabolic stability, good absorption/bioavailability, and either abundant cheap availability from a natural source or easy synthesis.

In theory, it is possible to reconstitute a “standardized propolis” of known composition for mechanistic studies, be they cellular or in vivo, although the optimal composition of such propolis might be a cause for some debate. However, as a potential treatment, this is highly unlikely to be commercially viable. It is therefore incumbent on the scientific community, at this point in time, to start selecting a limited panel of propolis-derived compounds with particular promise against a specific infectious agent, e.g., *Trypanosoma cruzi*, and take these as screening “hits” for further development. This will require a multidisciplinary consortium approach including medicinal chemistry for the development of structure–activity relationships (SAR), toxicology, pharmacokinetics/dynamics, biochemical parasitology for direct action studies, and immunology for the indirect effects of propolis. The screening of propolis fractions from various locations (with different vegetation) and the identification of the active compounds are of course by no means complete, but the development of new treatments from the knowledge accrued so far does not need to wait until the full catalog has been hoisted onto library shelves. Clearly, a good number of active compounds with apparent selectivity have been identified, and a “round table” of experts in medicinal chemistry, drug metabolism, drug delivery systems, and parasitology should be able to triage and select potential drug candidates from among them, as well as cost-effective ways to narrow the field further with standardized tests such as in vivo stability. This approach will give the field the clearest way forward toward genuine preclinical lead compounds for mechanistic, SAR, and extensive in vivo evaluations. It is only at this stage that serious partnerships with private sector pharmaceuticals or with multinational not-for-profit organization such as the Drugs for Neglected Diseases initiative (DNDi) become possible. A bundling of resources and coordination in efforts between research groups will be needed to start moving in the direction that all of us, according to the rationales stated in the introductions of our published papers, aspire to.

## Figures and Tables

**Table 1:** Some compounds isolated from African propolis between 2015 and 2020.

Name	Class of Compound	Country	Reference
Lupenone	Triterpenoid	Cameroon	[55]
α-Amyrin (**16**)	Triterpenoid	Cameroon/Nigeria	[15,55]
β-Amyrin	Triterpenoid	Cameroon	[55]
Methyl-3β,27-dihydroxycycloart-24-en-26-oate	Triterpenoid	Cameroon	[56]
Oleanolic acid	Triterpenoid	Cameroon	[57]
β-Amyrin acetate	Triterpenoid	Cameroon	[57]
Lupeol	Triterpenoid	Cameroon	[57]
Betulinic acid	Triterpenoid	Cameroon	[57]
Lupeol acetate	Triterpenoid	Cameroon	[57]
Cycloartanol	Cycloartane triterpene	Libya	[58]
Mangiferolic acid (**4**)	Cycloartane triterpene	Libya/Nigeria	[15,58]
Mangiferonic acid (**5**)	Cycloartane triterpene	Libya/Nigeria	[15,58]
Ambolic acid	Cycloartane triterpene	Libya	[58]
27-Hydroxymangiferonic acid (**6**)	Cycloartane triterpene	Libya	[58]
Ambonic acid (**15**)	Cycloartane triterpene	Nigeria	[15]
13-Epitorulosol	Diterpene	Libya	[58]
Acetylisocupressic acid (**7**)	Diterpene	Libya	[58]
Agathadiol (**8**)	Diterpene	Libya	[58]
Isocupressic acid (**9**)	Diterpene	Libya	[58]
Isoagatholal (**10**)	Diterpene	Libya	[58]
2-Hydroxy-8-prenylbiochanin A	Flavonoid	Cameroon	[57]
Taxifolin-3-acetyl-4′methyl ether (**11**)	Flavanoid	Libya	[58]
3,8-dihydroxy-9-methoxy-pterocarpan	Flavonoid	Nigeria	[39]
Astrapterocarpan	Flavonoid	Nigeria	[39]
Vesticarpan	Flavonoid	Nigeria	[39]
Vestitol (**21**)	Flavonoid	Nigeria	[15,39]
Broussonin B	Flavonoid	Nigeria	[39]
Calycosin	Flavonoid	Nigeria	[15]
Liquiritigenin (**45**)	Flavonoid	Nigeria	[15]
Pinocembrin	Flavonoid	Nigeria	[15]
Isosativan, (2′-hydroxy-7,4′-dimethoxyisoflavan)	Flavonoid	Nigeria	[37]
Medicarpin	Flavanoid	Nigeria	[39]
Pectolinarigenin	Flavonoid	Algeria	[59]
6,7-Dihydroxy-7,4′-dimethoxyflavone (Ladanein)	Flavonoid	Algeria	[59]
8-Prenylnaringenin (**20**)	Prenylated flavonoid	Nigeria	[15]
6-Prenylnaringenin	Prenylated flavonoid	Nigeria	[15]
Propolin D	Prenylated flavonoid	Nigeria	[15]
Macarangin	Prenylated flavonoid	Nigeria	[15]
Gerontoxanthone H (**12**)	Xanthone	Nigeria	[15]
6-Deoxy-γ-mangostin (**13**)	Xanthone	Nigeria	[15]
1,7-Dihydro-3-*O*-(3-methylbut-2-enyl)-8(3-methylbut-2-enyl) xanthone (**14**)	Xanthone	Nigeria	[15]
Demethylpiperitol (**1**)	Lignan	Libya	[59]
5′-Methoxypiperitol (**2**)	Lignan	Libya	[59]
Riverinol	Benzofuran	Nigeria	[15]
Triacontyl ρ-coumarate	Coumarin	Cameroon	[57]
Arachic/arachidic acid ethyl ester (PEN_4_)	Alkylphenol	Cameroon	[60]
Cardol	Alkylresorcinol	Libya/Cameroon	[55,58]
1′-*O*-Eicosanyl glycerol	Acylglycerol	Cameroon	[56]
Oleic acid	Fatty acid	Nigeria	[37]
Propyl stearate	Fatty acid ester	Nigeria	[37]
Hexatriacontanoic acid	Fatty acid	Cameroon	[55]
2′,3′-Dihydroxypropyltetraeicosanoate	Fatty acid	Cameroon	[57]

**Table 2:** Some compounds isolated from South American propolis between 2015 and 2020.

Name	Class of Compound	Country	Reference
β-Amyrin	Triterpenoid	Brazil	[61]
Glutinol	Triterpenoid	Brazil	[61]
Cycloart-24-en-3β-ol	Triterpenoid	Bolivia	[62,63]
Cycloart-24-en-3β,26-diol	Triterpenoid	Bolivia	[62,63]
24(*E*)-Cycloart-24-en-26-ol-3-one	Cycloartane triterpene	Bolivia	[62,63]
Cycloart-24-en-3-one	Cycloartane triterpene	Bolivia	[62,63]
Lupeol	Pentacyclic triterpene	Bolivia	[62,63]
Cycloartenone	Cycloartane triterpene	Bolivia	[62,63]
Liquiritigenin (**45**)	Flavonoid	Brazil	[61]
Isoliquiritigenin (**48**)	Flavonoid	Brazil	[61]
Formononetin (**46**)	Flavonoid	Brazil	[61]
Vestitol (**21**)	Flavonoid	Brazil	[61]
Neovestitol	Flavonoid	Brazil	[61]
Medicarpin	Flavonoid	Brazil	[61]
7-*O*-Neovestitol	Flavonoid	Brazil	[61]
3-*O*-Methylquercetin	Flavonoid	Brazil	[64]
3,6,4′-Trimethoxychrysin	Flavonoid	Brazil	[64]
3,6-Dimethoxyapigenin	Flavonoid	Brazil	[64]
6-Methoxykaempferol	Flavonoid	Brazil	[64]
6-Methoxyapigenin	Flavonoid	Brazil	[64]
5,7,4′-Trihydroxyflavanone (Naringenin)	Flavonoid	Ecuador	[65]
5,4′-Dihydroxy-7-methoxyflavanone (Sakuranetin)	Flavonoid	Ecuador	[65]
3,5,4′-Trihydroxy-7,3′-dimethoxyflavanone	Flavonoid	Ecuador	[65]
5,4′-Dihydroxy-7,3′-dimethoxyflavanon	Flavonoid	Ecuador	[65]
3,5,3′,4′-Tetrahydroxy-6,7-dimethoxy flavone (Eupatolitin)	Flavonoid	Ecuador	[65]
3,5,4′-Trihydroxy-7,3′-dimethoxy flavone (Rhamnazin)	Flavonoid	Ecuador	[65]
Pinocembrin	Flavonoid	Chile	[66]
Chrysin	Flavonoid	Chile	[66]
Kaempferol 3-methyl ether	Flavonoid	Bolivia	[62,63]
Kaempferol 7-*O*-methyl ether	Flavonoid	Bolivia	[62,63]
2-Phenoxychromone	Benzopyran derivative	Brazil	[64]
Cinnamic acid	Phenyl propanoid	Bolivia	[62,63]
3-Prenyl-*p*-coumaric acid (Drupanin)	Coumarin	Bolivia	[62,63]
Benzyl benzoate	Benzyl ester	Bolivia	[62,63]
Guttiferone E	Polyprenylated benzophenone	Brazil	[61]
Oblongifolin B	Polyprenylated benzophenone	Brazil	[61]
(*E*)-3-Hydroxy-1,7-diphenylhept-1-ene-5-acetate	Diarylheptanoid	Chile	[66]
(*E*)-5-Hydroxy-1,7-diphenylhept-1-ene-3-acetate	Diarylheptanoid	Chile	[66]

**Table 3:** Some compounds isolated from Asian propolis between 2015 and 2020.

Name	Class of Compound	Country	Reference
Mangiferolic acid	Cycloartane triterpenoid	Indonesia	[28]
Cycloartenol	Cycloartane triterpenoid	Indonesia	[28]
Mangferonic acid (**5**)	Cycloartane triterpenoid	Indonesia	[28]
Ambonic acid (**15**)	Cycloartane triterpenoid	Indonesia	[28]
Ambolic acid	Cycloartane triterpenoid	Indonesia	[28]
3-*O*-Acetyl ursolic acid	Triterpenoid	Thailand	[67]
Ocotillone I	Triterpenoid	Thailand	[67]
Ocotillone II	Triterpenoid	Thailand	[67]
Ursolic aldehyde	Triterpenoid	Thailand	[67]
Oleanolic aldehyde	Triterpenoid	Thailand	[67]
20-Hydroxy-24-dammaren-3-one	Triterpenoid	Malaysia	[68]
Dipterocarpol	Triterpenoid	Thailand	[67]
Cabralealactone	Triterpenoid	Thailand	[67]
Isocabralealactone	Triterpenoid	Thailand	[67]
β-Panasinsene	Sesquiterpene	Malaysia	[69]
α-Mangostin	Prenylated xanthone	Thailand	[70]
γ-Mangostin	Prenylated xanthone	Thailand	[70]
Cochinchinone T	Prenylated xanthone	Thailand	[70]
β-Mangostin	Prenylated xanthone	Thailand	[70]
Gartanin	Prenylated xanthone	Thailand	[70]
8-Deoxygartanin	Prenylated xanthone	Thailand	[70]
9-Hydroxycalabaxanthone	Prenylated xanthone	Thailand	[70]
Mangostanol	Prenylated xanthone	Thailand	[70]
Mangostanin	Xanthone	Thailand	[67]
Garcinone B	Xanthone	Thailand	[67]
Methylpinoresinol	Lignan	Thailand	[67]

**Table 4:** Some compounds isolated from Australian propolis between 2015 and 2020.

Name	Class of Compound	Country	Reference
3-Oxo-cycloart-24*E*-en-21,26-diol-21,26-diacetate	Triterpenoid	Pitcairn Island	[71]
3-Oxo-cycloart-24*E*-en-21,26-diol	Triterpenoid	Pitcairn Island	[71]
3-Oxo-cycloart-24*E*-en-21,26-diol-21-acetate	Triterpenoid	Pitcairn Island	[71]
3-Oxo-cycloart-24E-en-21,26-diol-26-acetate	Triterpenoid	Pitcairn Island	[71]
3-Oxo-cycloart-24-en-26-al	Triterpenoid	Pitcairn Island	[71]
7,8,18-Trihydroxyserrulat-14-ene	Diterpene	Australia	[72]
5,18-Epoxyserrulat-14-en-7,8-dione	Diterpene	Australia	[72]
(18*RS*)-5,18-Epoxyserrulat-14-en-8,18-diol	Diterpene	Australia	[72]
Abietinal	Diterpene	Pitcairn Island	[71]
Glyasperin	Flavonoid	Fiji Islands	[73]
(*E*)-4-(3-Methyl-2-buten-1-yl)-3,4′,5-trihydroxy-3′-methoxystilbene	Stilbene	Kangaroo Island	[74]
(*E*)-2-(3-Methyl-2-buten-1-yl)-3,4′,5-trihydroxystilbene (2-prenylresveratrol)	Stilbene	Kangaroo Island	[74]
(*E*)-2,4-Bis(3-methyl-2-buten-1-yl)-3,3′,4′,5-tetrahydroxystilbene	Stilbene	Kangaroo Island	[74]
(*E*)-2-(3-Methyl-2-buten-1-yl)-3-(3-methyl-2-butenyloxy)-3′,4′,5-trihydroxystilbene	Stilbene	Kangaroo Island	[74]
(*E*)-2,6-Bis(3-methyl-2-buten-1-yl)-3,3′,5,5′-tetrahydroxystilbene	Stilbene	Kangaroo Island	[74]
(*E*)-2,6-Bis-(3-methyl-2-buten-1-yl)-3,4′,5-trihydroxy-3′-methoxystilbene	Stilbene	Kangaroo Island	[74]
Tetragocarbone A	Phenol	Australia	[75]
Tetragocarbone B	Phenol	Australia	[75]
Solomonin B	Stilbene	Fiji Islands	[73]
Solomonin C	Stilbene	Fiji Islands	[73]
